# Towards Characterizing the Download Cost of Cache-Aided Private Updating †

**DOI:** 10.3390/e27080828

**Published:** 2025-08-04

**Authors:** Bryttany Stark, Ahmed Arafa, Karim Banawan

**Affiliations:** 1 Department of Electrical and Computer Engineering, University of North Carolina at Charlotte, Charlotte, NC 28223, USA; bryttany.stark@gmail.com; 2 Department of Electronics and Communications Engineering, The American University in Cairo, New Cairo 11835, Egypt; karim.banawan@aucegypt.edu; 3 Electrical Engineering Department, Faculty of Engineering, Alexandria University, Alexandria 21544, Egypt

**Keywords:** private information retrieval, coded caching, private updating, syndrome decoding

## Abstract

We consider the problem of privately updating a message out of *K* messages from *N* replicated and non-colluding databases where a user has an *outdated* version of the message ${\hat{W}}_{\theta }$ of length *L* bits that differ from the current version $W_{\theta }$ in at most *f* bits. The user also has a cache containing coded combinations of the *K* messages (with a pre-specified structure), which are unknown to the *N* databases (unknown prefetching). The cache *Z* contains *ℓ* linear combinations from all *K* messages in the databases with $r=\frac{l}{L}$ being the caching ratio. The user needs to retrieve $W_{\theta }$ correctly using a private information retrieval (PIR) scheme without leaking information about the message index θ to any individual database. Our objective is to jointly design the prefetching (i.e., the structure of said linear combinations) and the PIR strategies to achieve the least download cost. We propose a novel achievable scheme based on syndrome decoding where the cached linear combinations in *Z* are designed to be bits pertaining to the syndrome of $W_{\theta }$ according to a specific linear block code. We derive a general lower bound on the optimal download cost for 0≤r≤1, in addition to achievable upper bounds. The upper and lower bounds match for the cases when *r* is exceptionally low or high, or when K=3 messages for arbitrary *r*. Such bounds are derived by developing novel *cache-aided arbitrary message length* PIR schemes. Our results show a significant reduction in the download cost if $f<\frac{L}{2}$ when compared with downloading $W_{\theta }$ directly using typical cached-aided PIR approaches.

## 1. Introduction

The problem of private information retrieval (PIR), introduced by Chor et al. in [1], seeks to find the most efficient way for a user to privately retrieve a single message from a set of *K* messages from *N* fully replicated and non-communicating databases. PIR schemes are designed to download a *mixture* of all *K* messages, with the least number of overhead downloaded bits, such that no single database can infer the identity of the desired message. The user accomplishes this task by sending a query to each database. The databases respond truthfully to the submitted query with an answer string. The user can then reconstruct the desired message from jointly *decoding* the returned answer strings. Recently, the problem of PIR has received growing interest from the information and coding theory communities. The classical PIR problem is reformulated using information-theoretic measures in the seminal work of Sun–Jafar [2]. In there, the performance metric of the PIR scheme is the retrieval rate, which is the ratio of the number of the desired message symbols to the total number of downloaded bits. The supremum of this ratio is denoted by the PIR capacity, *C*. Sun and Jafar characterize the PIR capacity of the classical PIR model to be

(1) $$\begin{array}{c} C={\left(1+\frac{1}{N}+\frac{1}{N^{2}}+\cdots +\frac{1}{N^{K-1}}\right)}^{-1}. \end{array}$$

Following [2], the capacity (or its reciprocal, the normalized download cost) of many variations of the problem have been investigated; see [3,4,5,6,7,8,9,10,11,12,13,14,15,16,17], and the surveys in [18,19].

In all these works, the user is assumed to have no information about the desired message prior to retrieval. Thus, the queries are designed independently of the message contents. This is not always the case in practice. To see that, consider the following classical motivational example of PIR: in the stock market, investors need to privately retrieve some of the stock records since showing interest in a specific record may undesirably affect its value. PIR is a natural solution to this problem. Now, consider the case when an investor has already retrieved a specific stock record some time ago but this record has been changed. The investor needs to update the record at his/her side. A trivial solution to this problem is to reapply the original PIR scheme again. Nevertheless, this solution overlooks the fact that stock records are *correlated* in time. Another example arises in the context of private federated submodel learning [20], in which a user needs to retrieve the up-to-date desired submodel without leaking any information about its identity. The weights of each submodel are usually correlated in time as in the stock market example. In both examples, it is interesting to investigate whether or not the investor (user) can exploit the correlation between the outdated record (submodel) and its up-to-date counterpart to drive down the download cost. In this work, we focus our attention on a specific type of correlation, in which the up-to-date message is a distorted version of the outdated message according to a *Hamming distortion* measure.

The most closely related works to this problem are the PIR problems with side information, e.g., [21,22,23,24,25,26,27]. We also assume that the user has access to a private local cache containing equal portions of each message. Caching systems of this variety have been explored before in the PIR setting, e.g., [28,29], but not in conjunction with other forms of side information (outdated or updated). In the works regarding PIR with side information, the user has side information in the form of a subset of *undesired* messages, which are utilized to assist in privately retrieving the desired message. This is different from our setting, in which the user possesses side information in the form of an outdated *desired* message. Furthermore, these works differ from each other in whether the privacy of the side information should be maintained or not. This is different from our problem in which the identity of the desired and side information is the same, and therefore the privacy constraint in our problem is modified to reflect this fact.

In this work, we introduce the problem of *cache-aided private updating with unknown prefetching* for an *L*-bit length message out of a *K*-message library from *N* replicated and non-colluding databases. In this problem, the user has an *outdated* version ${\hat{W}}_{\theta }$ of the desired message θ, and wishes to update it to its up-to-date version $W_{\theta }$. Furthermore, the user has information about the *maximum* Hamming distance *f* between the up-to-date message and its outdated counterpart, i.e., the user possesses ${\hat{W}}_{\theta }$, which differs in *at most f* bits from the desired up-to-date message $W_{\theta }$. Based on ${\hat{W}}_{\theta }$ and *f*, the user needs to design a query set to reliably and privately decode the up-to-date version of the desired message $W_{\theta }$ with the least number of downloaded bits. Equivalently, the user needs to privately retrieve an *auxiliary* message that corresponds to the flipped bit positions in the desired message. Similar to the works of [30,31], we assume that the databases can construct a *mapping* from the original library of messages into a more appropriate form that can assist the user in the retrieval process (in this work, we assume that the databases are *semi-honest*, in the sense that they truthfully obey the retrieval process, but the databases are curious to learn the identity of the desired file). The user also has access to a private cache *Z* containing *ℓ* linear combinations of each message, with $r=\frac{l}{L}$ being the caching ratio. The structure of such linear combinations is pre-specified to facilitate the retrieval procedure. By jointly designing the prefetching (i.e., the structure of the aforementioned cache contents) and the updating procedures, we aim at characterizing the optimal download cost needed to update ${\hat{W}}_{\theta }$ to $W_{\theta }$ given *Z* without disclosing the desired message index θ to any of the databases for arbitrary *K*, *N*, *f*, *L*, and *r*.

To that end, we propose a novel achievable scheme that is based on the *syndrome decoding* idea introduced in [32], and adapt it to our setting to exploit the correlation between $W_{\theta }$ and ${\hat{W}}_{\theta }$. Hence, syndrome decoding is used to *compress* the desired message based on the user’s side information (i.e., the outdated message ${\hat{W}}_{\theta }$). More specifically, the databases apply a linear transformation to the stored library of messages using the parity check matrix of a linear block code with carefully chosen parameters. The existence of such a code can be readily inferred from the Gilbert–Varshamov and the Hamming bounds [33]. This transformation, in effect, maps the messages into their corresponding syndromes. Thus, the problem is reduced to retrieving the syndrome representation of the messages (i.e., the auxiliary messages) that comprises L¯=log2∑i=0fLi≤L bits, where *L* is the original message length.

In the case of r=0, we directly apply the PIR scheme in [34] to the auxiliary messages of length $\left\lceil \bar{L}\right\rceil$, which is optimal under the message length constraints. In the case where *r* satisfies $0<r\leq \frac{1}{1+N+N^{2}+\cdots +N^{K-1}}$ (denoted as very low *r*), $\frac{1}{1+N}\leq r\leq 1$ (denoted as very high *r*), we extend the PIR scheme in [34] to the cache-aided setting in [29], and develop a novel *cache-aided arbitrary message length* PIR scheme to solve our problem. We also present an achievable scheme for the mid-range *r*, satisfying $\frac{1}{1+N+N^{2}+\cdots +N^{K-1}}<r<\frac{1}{1+N}$, tailored for the case of K=3 messages, and discuss possible extensions for arbitrary *K* afterwards. Like with the r=0 case, we can then use this new cache-aided arbitrary message length scheme to download the auxiliary messages of length $\left\lceil \bar{L}\right\rceil$ with an effective caching ratio of $\tilde{r}=\frac{l}{\left\lceil \bar{L}\right\rceil }$. This is in effect a higher caching ratio than *r*, which in turns lead to a lower download cost as in [29]. For each of these cases, we confirm the validity of our proposed scheme by deriving a matching converse proof. Our converse proof is inspired by the converse proof of the cache-aided PIR problem with unknown and uncoded prefetching in [29], with the main difference being the fact that in addition to a private cache, the user has access to the outdated message ${\hat{W}}_{\theta }$, the index of which they wish to keep private. Consequently, we show that the optimal download cost is perfectly characterized for very high caching ratios, and is characterized within a maximum gap of only 2 bits otherwise. Notably, such a gap is 0 if $\bar{L}$ is an integer. This justifies the efficacy of using syndromes as a message-mixing technique in our setting. Furthermore, our results show that performing direct PIR on the original library of messages is strictly sub-optimal as long as the maximum Hamming distance $f<\frac{L}{2}$.

The rest of the paper is organized as follows. Our system model is described in Section 2. The main results are presented in Section 3, with the main converse proof following in Section 4, and the achievability proofs in Section 5 and Section 6. Section 7 includes a discussion on extending our achievability results, and the paper is concluded in Section 8.

## 2. System Model

We consider a classical PIR problem with *K* independent, uncoded, messages $W_{1},\ldots ,W_{K}$, with each message consisting of *L* independent and uniformly distributed bits. We have

(2) $$\begin{array}{cc} H(W_{i}) & =L,\;1\leq i\leq K, \end{array}$$

(3) $$\begin{array}{cc} H(W_{1},\ldots ,W_{K}) & =H\left(W_{1}\right)+\ldots +H\left(W_{K}\right). \end{array}$$

The *K* messages are stored in *N* replicated and non-communicating databases. The user (retriever) has a local copy of one of the messages whose index θ∈[K] is known to the user ([K] denotes the set {1,2,…,K}) but not the database (this is true if message θ, for example, has been previously obtained in a private manner). However, this message stored locally is *outdated*, and the user wishes to update it so that it is consistent with the copies in the databases without revealing to any of the databases what the message index is.

The user also has a local cache memory whose contents are denoted by a random variable *Z*. The cache is populated through a *prefetching phase* in which the user caches pre-specified linear combinations from each message $W_{i}$, i∈[K], with l<L bits (specifically, we consider the case when the prefetching and retrieval strategies can be jointly designed, i.e., we assume that the information source performing the prefetching may provide a linear combination of its content with any desired structure to assist the user in minimizing the download cost in the retrieval phase). Such linear combinations are represented by a matrix multiplication $W_{i}R_{i}$, where $R_{i}$ is of dimension L×l. Thus, we have

(4) $$\begin{array}{c} Z=[W_{1}R_{1},\;W_{2}R_{2},\;\cdots ,\;W_{K}R_{K}]. \end{array}$$

The explicit design of $R_{i}$, i∈[K] is specified along the lines of the achievability proof. We assume that the contents of the cache are *unknown* to the databases, as in, e.g., [21,27,29]. We define the *caching ratio* as

(5) $$\begin{array}{c} r=\frac{l}{L}. \end{array}$$

Observe that the number of cached bits pertaining to each message is equal to Lr. It now follows that

(6) $$\begin{array}{cc} H(Z) & =\sum_{i=1}^{K}H\left(W_{i}R_{i}\right)\leq KLr, \end{array}$$

(7) $$\begin{array}{cc} I(W_{i};Z) & =H(W_{i}R_{i})\leq Lr,\;1\leq i\leq K. \end{array}$$

The setting described above defines the *cache-aided private updating problem with unknown prefetching*.

Since each message is a string of *L* bits, the problem can be formulated as privately determining which subset of the message bits need to be flipped in order to fully update it. To model this, we use ${\hat{W}}_{\theta }$ to represent the locally stored outdated message, ${\bar{W}}_{\theta }$ to represent the subset of bit indices that need to be flipped, and *f* to represent the *maximum* Hamming distance between $W_{\theta }$ and ${\hat{W}}_{\theta }$ (clearly, f≥1 must hold; otherwise, there is no need to update ${\hat{W}}_{\theta }$). Therefore, in order to update message θ, the user needs to flip *at most f* bits, i.e., ${\bar{W}}_{\theta }$ takes a value out of ∑i=0fLi choices. We assume that such choices are uniformly distributed and independently realized from ${\hat{W}}_{\theta }$. Based on this model, the following holds:

(8) $$\begin{array}{cc} H\left(W_{\theta }\right)=H\left({\hat{W}}_{\theta }\right) & =L, \end{array}$$

(9) H(W¯θ)=log2∑i=0fLi≜L¯,

(10) $$\begin{array}{cc} H\left(W_{\theta }|{\hat{W}}_{\theta }\right)=H\left({\bar{W}}_{\theta }|{\hat{W}}_{\theta }\right) & =\bar{L}, \end{array}$$

(11) $$\begin{array}{cc} H({\bar{W}}_{\theta }|{\hat{W}}_{\theta },W_{\theta }) & =0, \end{array}$$

(12) $$|{\bar{W}}_{\theta }|\leq f\leq L,$$

where |·| denotes cardinality. We assume that the maximum Hamming distance *f* between the outdated and updated message is known to the user. By (9), one can see that $\left\lceil \bar{L}\right\rceil$ bits should be sufficient to update ${\hat{W}}_{\theta }$. Hence, one can set a maximum value on the number of cached bits from each message as follows (in case the number of cached bits is greater than this bound in (13), the extra bits can be ignored by the user):

(13) $$\begin{array}{c} l\leq \left\lceil \bar{L}\right\rceil . \end{array}$$

In order to retrieve $W_{\theta }$, the user sends a set of queries $Q_{1}^{\left[\theta \right]},\ldots ,Q_{N}^{\left[\theta \right]}$ to the *N* databases to efficiently obtain ${\bar{W}}_{\theta }$. The queries are generated according to ${\hat{W}}_{\theta }$, *f*, and *Z*, and are jointly independent of the realizations of the [K]∖{θ} messages and ${\bar{W}}_{\theta }$ given ${\hat{W}}_{\theta }$. Therefore we have (we use the notation $x_{S}$ to denote the collection of $\left\{x_{i},\;i\in S\right\}$)

(14) $$\begin{array}{c} I\left(W_{\left[K]\setminus \{\theta \right\}},{\bar{W}}_{\theta };Q_{1:N}^{\left[\theta \right]}|{\hat{W}}_{\theta },Z\right)=0. \end{array}$$

Upon receiving the query $Q_{n}^{\left[\theta \right]}$, the *n*th database replies with an answering string $A_{n}^{\left[\theta \right]}$, which is a function of $Q_{n}^{\left[\theta \right]}$ and all the *K* messages stored. Therefore, ∀θ∈[K],∀n∈[N], we have

(15) $$\begin{array}{c} H\left(A_{n}^{\left[\theta \right]}|Q_{n}^{\left[\theta \right]},W_{1:K}\right)=0. \end{array}$$

To ensure that individual databases do not know which message is being updated, we need to satisfy the following *privacy constraint*, ∀n∈[N],∀k∈[K]:

(16) $$\begin{array}{c} \;\left(\;Q_{n}^{\left[1\right]},A_{n}^{\left[1\right]},{\hat{W}}_{1},W_{1:K}\;\right)∼\left(\;Q_{n}^{\left[k\right]},A_{n}^{\left[k\right]},{\hat{W}}_{k},W_{1:K}\;\right)\;, \end{array}$$

where ∼ denotes statistical equivalence. After receiving the answering strings $A_{1:N}^{\left[\theta \right]}$ from all the *N* databases, the user needs to decode the desired information $W_{\theta }$ with no uncertainty, satisfying the following *correctness constraint*:

(17) $$\begin{array}{c} H\left(W_{\theta }|A_{1:N}^{\left[\theta \right]},Q_{1:N}^{\left[\theta \right]},{\hat{W}}_{\theta },Z\right)=0. \end{array}$$

**Figure 1:**
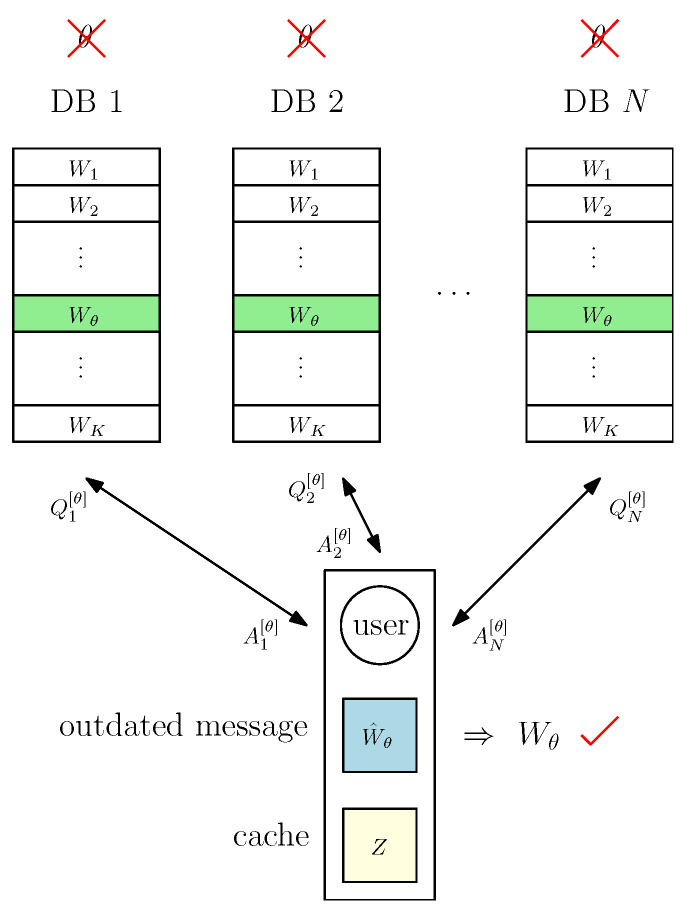
Cache-aided private updating with unknown prefetching system model.

The overall system model is depicted in Figure 1. We also include a list of notation with their definitions in Table 1 for ease of presentation.

For fixed *N*, *K*, *f*, and *r*, a pair $\left(\bar{D},L\right)$ is *achievable* if there exists a cache-aided private updating with unknown prefetching scheme for messages of length *L* bits long satisfying the privacy constraint (16) and the correctness constraint (17). In this pair, $\bar{D}$ represents the expected number of downloaded bits received from the *N* databases independently via the answering strings $A_{1:N}^{\left[k\right]}$, i.e.,

(18) $$\begin{array}{c} \bar{D}=\sum_{n=1}^{N}H\left(A_{n}^{\left[\theta \right]}\right). \end{array}$$

*Our goal is to characterize the optimal download cost ${\bar{D}}_{L}$ for the cache-aided private updating problem with unknown prefetching for fixed arbitrary L, N, K, f, and r*. That is, we solve for

(19) $$\begin{array}{c} {\bar{D}}_{L}=\min\left\{\bar{D}:\left(\bar{D},L\right)\mathrm{is}\;\mathrm{achievable}\right\}. \end{array}$$

Clearly, the user can ignore its outdated message ${\hat{W}}_{\theta }$ and re-download the whole new message $W_{\theta }$ using standard cache-aided PIR schemes [2,29]. Our main result, however, shows that we can use ${\hat{W}}_{\theta }$ to do strictly better.

## 3. Main Results

Our first result characterizes a converse bound for the optimal download cost ${\bar{D}}_{L}$ for general *N*, *K*, *f*, and *r*.

**Theorem 1**  (Converse)**.** *In the cache-aided private updating problem with unknown prefetching, the optimal download cost is lower bounded by*

(20) $$\begin{array}{c} {\bar{D}}_{L}\geq \left\lceil \max_{i\in \{2,\ldots ,K+1\}}\;\left(\bar{L}\;-\;Lr\right)\;\sum_{j=0}^{K+1-i}\;\;\frac{1}{N^{j}}\;-\;Lr\;\sum_{j=0}^{K-i}\;\frac{K\;+\;1\;-\;i\;-\;j}{N^{j}}\right\rceil , \end{array}$$

*with $\bar{L}$ defined in (9).*

The proof of Theorem 1 is provided in Section 4.

For our next result, we characterize an achievability bound for specific values of the caching ratios, and otherwise general *L*, *N*, *K*, and *f*. Before we present our result, we need to introduce some notation. Specifically, as in [29], for s∈{1,2,…,K−1}, we define a caching ratio $r_{s}$ as

(21) rs=K−2s−1K−2s−1+∑i=0K−1−sK−1s+i(N−1)iN.

Now, we say that a caching ratio *r* is *very low* if $0\leq r\leq r_{1}=\frac{1}{1+N+N^{2}+\cdots +N^{K-1}}$, *very high* if $r_{K-1}=\frac{1}{1+N}\leq r\leq 1$, and *mid-range* otherwise. We are now ready to present our first achievability result.

**Theorem 2**  (Very Low and Very High Achievability)**.** *In the cache-aided private updating problem with unknown prefetching, for very low caching ratios, the optimal download cost is upper bounded by*

(22) $$\begin{array}{c} {\bar{D}}_{L}\leq \left\lceil \left(\left\lceil \bar{L}\right\rceil \;-\;Lr\right)\cdot \;\sum_{i=0}^{K-1}\frac{1}{N^{i}}-Lr\cdot \;\sum_{i=0}^{K-2}\frac{K-1-i}{N^{i}}\right\rceil , \end{array}$$

*and for very high caching ratios, the optimal download cost is upper bounded by*

(23) $$\begin{array}{c} {\bar{D}}_{L}\leq \left\lceil \bar{L}\right\rceil -Lr, \end{array}$$

*with $\bar{L}$ defined in (9).*

The proof of Theorem 2 is provided in Section 5.

Combining the achievability bounds in Theorem 2 with the converse bound in Theorem 1, we obtain a fairly tight, up to a ceiling difference of $\bar{L}$, characterization of the optimal download cost ${\bar{D}}_{L}$ for very low and very high caching ratios. This is stated in the following corollary.

**Corollary 1.**  *In the cache-aided private updating problem with unknown prefetching, for very low caching ratios, we have*

(24) $$\begin{array}{cc} \; & \left\lceil \left(\bar{L}\;-\;Lr\right)\sum_{j=0}^{K-1}\frac{1}{N^{j}}\;-\;Lr\sum_{j=0}^{K-2}\frac{K-1-j}{N^{j}}\right\rceil \leq {\bar{D}}_{L}\leq \left\lceil \left(\left\lceil \bar{L}\right\rceil \;-\;Lr\right)\sum_{j=0}^{K-1}\frac{1}{N^{j}}\;-\;Lr\sum_{j=0}^{K-2}\frac{K-1-j}{N^{j}}\right\rceil , \end{array}$$

*and for very high caching ratios, we have*

(25) $$\begin{array}{c} {\bar{D}}_{L}=\left\lceil \bar{L}\right\rceil -Lr \end{array}$$

**Proof.**  The right-hand side inequality of (24) is given directly by Theorem 2. By choosing i=2 in (20), we obtain the left-hand side inequality in (24). Similarly, by choosing i=K−1 in (20), we obtain the result in (25) (note that Lr is an integer, and so in this case, the converse and achievability bounds match). This concludes the proof. □

We now have the following remarks.

**Remark 1.**  *The result in Corollary 1 generalizes our preliminary work on the private updating problem with no caching involved [35]. Specifically, plugging in r=0 in Corollary 1 directly gives ([35], Theorem 1).*

**Figure 2:**
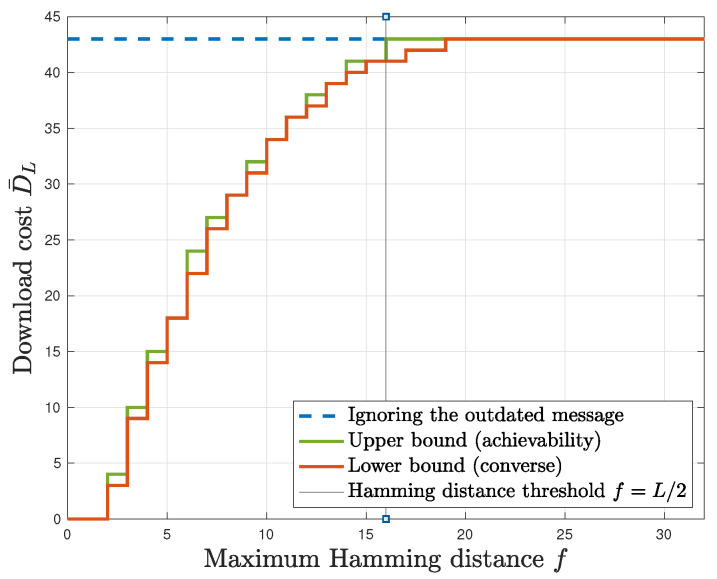
Download cost of cache-aided private updating with unknown prefetching with L=32 bits, N=2 databases, K=3 messages, and $r=\frac{1}{10}$ caching ratio (Corollary 1’s results for the very low caching ratio).

**Remark 2.**  *Consider the result in (24). From (9) and (12), it follows that $\left\lceil \bar{L}\right\rceil =L$ for all values of $f\geq \frac{L}{2}$, and that $\left\lceil \bar{L}\right\rceil <L$ for all values of $f<\frac{L}{2}$ (this can be readily shown using the binomial theorem; details are in Appendix A). Combining this with the results in ([29], Corollary 2) (which is the analog of our result in case the user does not have an outdated message), this means that there is a* Hamming distance threshold *of $\frac{L}{2}$ beyond which there is no advantage to using a private updating strategy, and below which there will always be some savings in download cost. This can be seen in Figure 2, where we also note that the non-linearity of the upper and lower bounds are a result of the ceiling functions that appear in these bounds.*

**Remark 3.**  *If L and f are such that $\bar{L}=\left\lceil \bar{L}\right\rceil$, then the upper and lower bounds in (24) match. We will see that this holds if a* perfect code *(a code that attains the Hamming bound with equality [33]) by which the queries are sent exists (cf. Section 5). Otherwise, if $\bar{L}<\left\lceil \bar{L}\right\rceil$, one can show using similar arguments as in ([34], Section 7.2) that the two bounds are within 2 bits for N≥2 databases.*

Next, we have the following achievability result regarding mid-range caching ratios.

**Theorem 3**  (Mid-Range Achievability)**.** *In the cache-aided private updating problem with unknown prefetching with K=3 messages, for mid-range effective caching ratios, the optimal download cost is upper bounded by*

(26) $$\begin{array}{c} {\bar{D}}_{L}\leq \left\lceil \left(\left\lceil \bar{L}\right\rceil -Lr\right)\left(1+\frac{1}{N}\right)-Lr\right\rceil \end{array}$$

*with $\bar{L}$ defined in (9).*

The proof of Theorem 3 is provided in Section 6. In Section 7, we include a discussion on extending the above achievability result for arbitrary *K*.

Combining the mid-range achievability bound in Theorem 3 and the converse bound in Theorem 1 for i=K, we characterize the optimal download cost for ${\bar{D}}_{L}$ for mid-range caching ratios when K=3. Furthermore, combining this characterization with the result of Corollary 1 gives a complete characterization of ${\bar{D}}_{L}$ when K=3 for *any* caching ratio. To this end, we define the *K=3 converse* bound ${\bar{D}}_{K=3}\left(r\right)$ and the *K=3 achievability* bound ${\bar{D}}^{K=3}\left(r\right)$ to express this characterization:

(27) $$\begin{array}{c} {\bar{D}}_{K=3}\left(r\right)=\left\{\begin{array}{cc} \;\left\lceil \;\left(\bar{L}\;-\;Lr\right)\;\cdot \;\sum_{i=0}^{2}\frac{1}{N^{i}}\;-\;Lr\;\cdot \;\sum_{i=0}^{1}\frac{2\;-\;i}{N^{i}}\right\rceil ,\;\; & \;\mathrm{if}\;0\leq r\leq r_{1};\\ \;\left\lceil \left(\bar{L}-Lr\right)\left(1+\frac{1}{N}\right)-Lr\right\rceil , & \;\mathrm{if}\;r_{1}\leq r\leq r_{2};\\ \;\left\lceil \bar{L}\right\rceil -Lr, & \;\mathrm{if}\;r_{2}\leq r\leq 1. \end{array}\right. \end{array}$$

(28) $$\begin{array}{cc} \; & {\bar{D}}^{K=3}\left(r\right)=\left\{\begin{array}{cc} \;\left\lceil \;\left(\left\lceil \bar{L}\right\rceil \;-\;Lr\right)\;\cdot \;\;\sum_{i=0}^{2}\;\frac{1}{N^{i}}\;-\;Lr\;\cdot \;\;\sum_{i=0}^{1}\;\frac{2\;-\;i}{N^{i}}\right\rceil ,\;\;\;\; & \;\mathrm{if}\;0\leq r\leq r_{1};\\ \;\left\lceil \left(\left\lceil \bar{L}\right\rceil -Lr\right)\left(1+\frac{1}{N}\right)-Lr\right\rceil , & \;\mathrm{if}\;r_{1}\leq r\leq r_{2};\\ \;\left\lceil \bar{L}\right\rceil -Lr, & \;\mathrm{if}\;r_{2}\leq r\leq 1. \end{array}\right. \end{array}$$

We have now proved the following corollary.

**Corollary 2**  (K=3 Characterization)**.** *In the cache-aided private updating problem with unknown prefetching where K=3, for any caching ratio, we have*

(29) $$\begin{array}{c} {\bar{D}}_{K=3}\left(r\right)\leq {\bar{D}}_{L}\leq {\bar{D}}^{K=3}\left(r\right) \end{array}$$

## 4. Proof of Theorem 1: Converse

In this section, we derive the general (converse) lower bound for the download cost in Theorem 1. To do so, we prove two useful lemmas, analogues to their counterparts in the cache-aided PIR setting of [29], for the case of our cache-aided private updating problem. The two lemmas are then combined to prove the general lower bound. The key difference between our lemmas and those in [29] is that in addition to some uniform portion of each message being cached, the user is given an outdated message ${\hat{W}}_{\theta }$, requiring careful handling of the correlation between $W_{\theta }$ and ${\hat{W}}_{\theta }$.

**Lemma 1**  (Interference Lower Bound)**.** *In the cache-aided private updating problem with unknown prefetching, the interference from undesired messages within the answering strings, $\bar{D}-\left(\bar{L}-Lr\right)$, satisfies*

(30) $$\begin{array}{c} \bar{D}-\left(\bar{L}-Lr\right)\geq I\left(W_{k:K};Q_{1:N}^{\left[k-1\right]},A_{1:N}^{\left[k-1\right]}|W_{1:k-1},{\hat{W}}_{k-1},Z\right) \end{array}$$

*for all k∈{2,…,K}.*

**Proof.**  We start with the right-hand side of (30),

$$\begin{array}{cc} \; & I(W_{k:K};Q_{1:N}^{\left[k-1\right]},A_{1:N}^{\left[k-1\right]}|W_{1:k-1},{\hat{W}}_{k-1},Z) \end{array}$$

(31) $$\begin{array}{cc} \; & =I\left(W_{k:K};Q_{1:N}^{\left[k-1\right]},A_{1:N}^{\left[k-1\right]},W_{k-1}|W_{1:k-2},{\hat{W}}_{k-1},Z\right)-I\left(W_{k:K};W_{k-1}|W_{1:k-2},{\hat{W}}_{k-1},Z\right) \end{array}$$

$$\begin{array}{cc} \; & =I\left(W_{k:K};Q_{1:N}^{\left[k-1\right]},A_{1:N}^{\left[k-1\right]}|W_{1:k-2},{\hat{W}}_{k-1},Z\right)+I\left(W_{k:K};W_{\;k-1}|Q_{1:N}^{\left[k-1\right]},A_{1:N}^{\left[k-1\right]},W_{\;1:k-2},{\hat{W}}_{\;k-1},Z\right) \end{array}$$

(32) $$\begin{array}{cc} \; & \overset{\left(17\right)}{=}I\left(W_{k:K};Q_{1:N}^{\left[k-1\right]},A_{1:N}^{\left[k-1\right]}|W_{1:k-2},{\hat{W}}_{k-1},Z\right) \end{array}$$

(33) $$\begin{array}{cc} \; & \overset{\left(14\right)}{=}I\left(W_{k:K};A_{1:N}^{\left[k-1\right]}|Q_{1:N}^{\left[k-1\right]},W_{1:k-2},{\hat{W}}_{k-1},Z\right) \end{array}$$

(34) $$\begin{array}{cc} \; & =H\left(A_{1:N}^{\left[k-1\right]}|Q_{1:N}^{\left[k-1\right]},W_{1:k-2},{\hat{W}}_{k-1},Z\right)-H\left(A_{1:N}^{\left[k-1\right]}|Q_{1:N}^{\left[k-1\right]},W_{1:k-2},W_{k:K},{\hat{W}}_{k-1},Z\right) \end{array}$$

(35) $$\begin{array}{cc} \; & \overset{\left(17\right)}{=}H\left(A_{1:N}^{\left[k-1\right]}|Q_{1:N}^{\left[k-1\right]},W_{1:k-2},{\hat{W}}_{k-1},Z\right)-\;H\left(A_{1:N}^{\left[k-1\right]},W_{\;k-1}|Q_{1:N}^{\left[k-1\right]},W_{\;1:k-2},W_{\;k:K},{\hat{W}}_{\;k-1},Z\right) \end{array}$$

(36) $$\begin{array}{cc} \; & \leq H\left(A_{1:N}^{\left[k-1\right]}|Q_{1:N}^{\left[k-1\right]},W_{1:k-2},{\hat{W}}_{k-1},Z\right)-H\left(W_{k-1}|Q_{1:N}^{\left[k-1\right]},W_{1:k-2},W_{k:K},{\hat{W}}_{k-1},Z\right) \end{array}$$

(37) $$\begin{array}{cc} \; & \overset{\left(14\right)}{=}H\left(A_{1:N}^{\left[k-1\right]}|Q_{1:N}^{\left[k-1\right]},W_{1:k-2},{\hat{W}}_{k-1},Z\right)-H\left(W_{k-1}|{\hat{W}}_{k-1},Z\right) \end{array}$$

(38) $$\begin{array}{cc} \; & \overset{(18),\left(3\right)}{\leq }\bar{D}-H\left(W_{k-1}|{\hat{W}}_{k-1},W_{k-1}R_{k-1}\right) \end{array}$$

(39) $$\begin{array}{cc} \; & =\bar{D}-\left(H\left(W_{k-1},W_{k-1}R_{k-1}|{\hat{W}}_{k-1}\right)-H\left(W_{k-1}R_{k-1}|{\hat{W}}_{k-1}\right)\right) \end{array}$$

(40) $$\begin{array}{cc} \; & =\bar{D}-\left(H\left(W_{k-1}|{\hat{W}}_{k-1}\right)+H\left(W_{k-1}R_{k-1}|{\hat{W}}_{k-1},W_{k-1}\right)-H\left(W_{k-1}R_{k-1}|{\hat{W}}_{k-1}\right)\right) \end{array}$$

(41) $$\begin{array}{cc} \; & \overset{\left(10),(7\right)}{\leq }\bar{D}-\left(\bar{L}-Lr\right). \end{array}$$

This concludes the proof. □

Note that if privacy was not a constraint, then $\bar{D}=\bar{L}-Lr$ and the interference from undesired messages would be non-existent. However, when the privacy constraint is present, $\bar{D}-\left(\bar{L}-Lr\right)$ characterizes the number of bits that will be downloaded and used as side information to preserve privacy from the databases in a given scheme.

**Lemma 2**  (Induction Lemma)**.** *For all k∈{2,…,K}, the mutual information term in Lemma 1 can be inductively lower bounded as*

$$\begin{array}{cc} \; & I\left(W_{k:K};Q_{1:N}^{\left[k-1\right]},A_{1:N}^{\left[k-1\right]}|W_{1:k-1},{\hat{W}}_{k-1},Z\right) \end{array}$$

(42) $$\begin{array}{cc} \; & \;\geq \frac{1}{N}I\left(W_{k+1:K};Q_{1:N}^{\left[k\right]},A_{1:N}^{\left[k\right]}|W_{1:k},{\hat{W}}_{k},Z\right)+\frac{\bar{L}-Lr}{N}-\left(K-k+1\right)Lr. \end{array}$$

**Proof.**  We start with the left-hand side of (42),

$$\begin{array}{cc} \; & I(W_{k:K};Q_{1:N}^{\left[k-1\right]},A_{1:N}^{\left[k-1\right]}|W_{1:k-1},{\hat{W}}_{k-1},Z) \end{array}$$

(43) $$\begin{array}{cc} \; & =I\left(W_{k:K};Q_{1:N}^{\left[k-1\right]},A_{1:N}^{\left[k-1\right]},Z,{\hat{W}}_{k-1}|W_{1:k-1}\right)-I\left(W_{k:K};Z,{\hat{W}}_{k-1}|W_{1:k-1}\right) \end{array}$$

$$\begin{array}{cc} \; & =I\left(W_{k:K};Q_{1:N}^{\left[k-1\right]},A_{1:N}^{\left[k-1\right]}|W_{1:k-1}\right)+I\left(W_{k:K};Z,{\hat{W}}_{k-1}|W_{1:k-1},Q_{1:N}^{\left[k-1\right]},A_{1:N}^{\left[k-1\right]}\right) \end{array}$$

(44) $$\begin{array}{cc} \; & \;-I(W_{k:K};Z,{\hat{W}}_{k-1}|W_{1:k-1}) \end{array}$$

(45) $$\begin{array}{cc} \; & \geq I\left(W_{k:K};Q_{1:N}^{\left[k-1\right]},A_{1:N}^{\left[k-1\right]}|W_{1:k-1}\right)-I\left(W_{k:K};Z,{\hat{W}}_{k-1}|W_{1:k-1}\right). \end{array}$$

Now, for the first term in (45), we have

(46) $$\begin{array}{cc} \; & I(W_{k:K};Q_{1:N}^{\left[k-1\right]},A_{1:N}^{\left[k-1\right]}|W_{1:k-1}) \end{array}$$

(47) $$\begin{array}{cc} \; & \geq \frac{1}{N}\sum_{n=1}^{N}I\left(W_{k:K};Q_{n}^{\left[k-1\right]},A_{n}^{\left[k-1\right]}|W_{1:k-1}\right) \end{array}$$

(48) $$\begin{array}{cc} \; & \overset{\left(16\right)}{=}\frac{1}{N}\sum_{n=1}^{N}I\left(W_{k:K};Q_{n}^{\left[k\right]},A_{n}^{\left[k\right]}|W_{1:k-1}\right) \end{array}$$

(49) $$\begin{array}{cc} \; & =\frac{1}{N}\sum_{n=1}^{N}I\left(W_{k:K};A_{n}^{\left[k\right]}|W_{1:k-1},Q_{n}^{\left[k\right]}\right) \end{array}$$

(50) $$\begin{array}{cc} \; & \overset{\left(15\right)}{=}\frac{1}{N}\sum_{n=1}^{N}H\left(A_{n}^{\left[k\right]}|W_{1:k-1},Q_{n}^{\left[k\right]}\right) \end{array}$$

(51) $$\begin{array}{cc} \; & \geq \frac{1}{N}\sum_{n=1}^{N}H\left(A_{n}^{\left[k\right]}|W_{1:k-1},{\hat{W}}_{k},Z,Q_{1:N}^{\left[k\right]},A_{1:n-1}^{\left[k\right]}\right) \end{array}$$

(52) $$\begin{array}{cc} \; & \overset{\left(15\right)}{=}\frac{1}{N}\sum_{n=1}^{N}I\left(W_{\;k:K};A_{n}^{\left[k\right]}|W_{\;1:k-1},{\hat{W}}_{\;k},Z,Q_{1:N}^{\left[k\right]},A_{1:n-1}^{\left[k\right]}\right) \end{array}$$

(53) $$\begin{array}{cc} \; & =\frac{1}{N}I\left(W_{k:K};A_{1:N}^{\left[k\right]}|W_{1:k-1},{\hat{W}}_{k},Z,Q_{1:N}^{\left[k\right]}\right) \end{array}$$

(54) $$\begin{array}{cc} \; & \overset{\left(14\right)}{=}\frac{1}{N}I\left(W_{k:K};Q_{1:N}^{\left[k\right]},A_{1:N}^{\left[k\right]}|W_{1:k-1},{\hat{W}}_{k},Z\right) \end{array}$$

(55) $$\begin{array}{cc} \; & \overset{\left(17\right)}{=}\frac{1}{N}I\left(W_{k:K};W_{k},Q_{1:N}^{\left[k\right]},A_{1:N}^{\left[k\right]}|W_{1:k-1},{\hat{W}}_{k},Z\right) \end{array}$$

(56) $$\begin{array}{cc} \; & =\frac{1}{N}I\left(W_{k:K};Q_{1:N}^{\left[k\right]},A_{1:N}^{\left[k\right]}|W_{1:k},{\hat{W}}_{k},Z\right)+\frac{1}{N}I\left(W_{k:K};W_{k}|W_{1:k-1},{\hat{W}}_{k},Z\right) \end{array}$$

(57) $$\begin{array}{cc} \; & =\frac{1}{N}I\left(W_{\;k:K};Q_{1:N}^{\left[k\right]},A_{1:N}^{\left[k\right]}|W_{\;1:k},{\hat{W}}_{\;k},Z\right)\;+\;\frac{1}{N}H\left(W_{k}|{\hat{W}}_{k},Z\right) \end{array}$$

(58) $$\begin{array}{cc} \; & \overset{\left(10),(7\right)}{\geq }\frac{1}{N}I\left(W_{k+1:K};Q_{1:N}^{\left[k\right]},A_{1:N}^{\left[k\right]}|W_{1:k},{\hat{W}}_{k},Z\right)+\frac{\bar{L}-Lr}{N}. \end{array}$$

Note that (58) follows from a similar argument in Lemma 1 starting at (37). Next, for the second term in (45), we have

$$\begin{array}{cc} \; & I(W_{k:K};Z,{\hat{W}}_{k-1}|W_{1:k-1}) \end{array}$$

(59) $$\begin{array}{cc} \; & =H\left(W_{k:K}|W_{1:k-1}\right)-H\left(W_{k:K}|W_{k-1},Z,{\hat{W}}_{k-1}\right) \end{array}$$

(60) $$\begin{array}{cc} \; & =(K-k+1)L-(K-k+1)L(1-r) \end{array}$$

(61) $$\begin{array}{cc} \; & =(K-k+1)Lr \end{array}$$

Combining the above results concludes the proof. □

We now apply the result of Lemma 2 recursively on that of Lemma 1 to get the general lower bound through the following series of inequalities:

(62) $$\begin{array}{cc} \bar{D} & \overset{\left(30\right)}{\geq }\;\;\left(\bar{L}\;-\;Lr\right)\;+\;I\left(W_{\;k:K};Q_{\;1:N}^{\left[k-\;1\right]},A_{1:N}^{\left[k-\;1\right]}|W_{\;1:k-1},{\hat{W}}_{\;1},Z\right) \end{array}$$

$$\begin{array}{cc} \; & \overset{\left(42\right)}{\geq }\left(\bar{L}-Lr\right)+\frac{\bar{L}-Lr}{N}\\ \; & \;+\frac{1}{N}I\left(W_{k+1:K};Q_{1:N}^{\left[k\right]},A_{1:N}^{\left[k\right]}|W_{1:k},{\hat{W}}_{k},Z\right) \end{array}$$

(63) $$\begin{array}{cc} \; & \;-(K-k+1)Lr \end{array}$$

$$\begin{array}{cc} \; & \overset{\left(42\right)}{\geq }\left(\bar{L}-Lr\right)+\frac{\bar{L}-Lr}{N}+\frac{\bar{L}-Lr}{N^{2}}\\ \; & \;+\frac{1}{N^{2}}I\left(W_{k+2:K};Q_{1:N}^{\left[k+1\right]},A_{1:N}^{\left[k+1\right]}|W_{1:k+1},{\hat{W}}_{k+1},Z\right) \end{array}$$

(64) $$\begin{array}{cc} \; & \;-\left(K-k+1\right)Lr+\frac{(K-k+2)Lr}{N} \end{array}$$

(65) $$\begin{array}{cc} \; & \overset{\left(42\right)}{\geq }\ldots \end{array}$$

(66) $$\begin{array}{cc} \; & =\left(\bar{L}-Lr\right)\sum_{j=0}^{K+1-k}\frac{1}{N^{j}}-Lr\sum_{j=0}^{K-k}\frac{K+1-k-j}{N^{j}} \end{array}$$

Next, since the bound in (66) is valid for arbitrary *k*, it is still valid for *k* corresponding to the maximum possible lower bound, i.e., (66) gives *K* intersecting line segments, therefore, the download cost $\bar{D}$ is lower bounded by their maximum value

(67) $$\begin{array}{c} \bar{D}\geq \max_{i\in \{2,\ldots ,K+1\}}\left(\bar{L}\;-\;Lr\right)\;\sum_{j=0}^{K+1-i}\frac{1}{N^{j}}\;-\;Lr\sum_{j=0}^{K-i}\frac{K\;+\;1\;-\;i\;-\;j}{N^{j}}. \end{array}$$

Since (67) lower bounds the download cost $\bar{D}$ for *any* cache-aided private updating with unknown prefetching scheme, it also lower bounds the download cost of the *optimal* private updating scheme ${\bar{D}}_{L}$. Finally, since ${\bar{D}}_{L}$ is an integer, we take the ceiling of (67) to get (20).

This concludes the converse proof.

## 5. Proof of Theorem 2: Achievability for Very Low and Very High Caching Ratios

Our achievability scheme makes use of the correlation between $W_{\theta }$ and ${\hat{W}}_{\theta }$ through the knowledge of their maximum Hamming distance *f* in order to reduce the download cost. This approach is related to the problem tackled in [32] (without privacy constraints), in which a source is compressed given that it is correlated with some side information that is available only at the decoder. The retrieving user represents the decoder in our case, with side information ${\hat{W}}_{\theta }$. By the Slepian–Wolf coding theorem [36], one can noiselessly compress the source $W_{\theta }$ at the rate of $H\left(W_{\theta }|{\hat{W}}_{\theta }\right)=\bar{L}$. The *compressed* source is treated as a *new message* to be downloaded using a PIR scheme, as opposed to downloading the whole message $W_{\theta }$. Such a scheme, however, has a message length constraint (unlike most of the PIR works in the literature). For that reason, we leverage tools from the PIR scheme with an arbitrary message length in [34], and extend them to work in the caching setting at hand, to accomplish our task.

While our achievability schemes make use of the local cache *Z*, we will first give some motivating examples without the user having knowledge of *Z*, which represents the case r=0 tackled in our preliminary work [35].

### 5.1. Motivating Examples Without Caching

#### 5.1.1. L=3, N=2, K=2, f=1, and r=0

In this example, we have $\bar{L}=\log_{2}\left(1+3\right)=2$, and C=2/3 (from (1)). Setting r=0 in (22), we need to show that $\bar{D}=\left\lceil \left\lceil \bar{L}\right\rceil /C\right\rceil =3$ bits is achievable. We first start by constructing a [3,1,3] linear block code, which is in this case a repetition code with generator matrix G and parity check matrix H given by

(68) $$\begin{array}{c} G=\left[\begin{array}{ccc} 1 & 1 & 1 \end{array}\right],\;H=\left[\begin{array}{ccc} 1 & 1 & 0\\ 1 & 0 & 1 \end{array}\right]. \end{array}$$

Note that such code is capable of correcting at most f=1 error. The syndromes associated with this code are s∈{00,01,10,11}. Observe that the length of s is exactly $\left\lceil \bar{L}\right\rceil$.

Instead of requesting $W_{\theta }$, the user retrieves the index of the coset in which $W_{\theta }$ resides in the code’s standard array. That is, its corresponding syndrome

(69) $$\begin{array}{c} s_{\theta }=W_{\theta }H^{T}. \end{array}$$

The user then compares ${\hat{W}}_{\theta }$ to all the words in that coset, and decodes $W_{\theta }$ as the one closest in Hamming distance. This is guaranteed to yield the unique correct message [32]. Therefore, the syndrome $s_{\theta }$ efficiently represents the flipped bits’ indices ${\bar{W}}_{\theta }$, and one is able to reduce the effective message length from L=3 to $\left\lceil \bar{L}\right\rceil =2$ by dealing with the syndrome $s_{\theta }$ instead of $W_{\theta }$.

Let $W_{1}=\left[a_{1},a_{2},a_{3}\right]$, and $W_{2}=\left[b_{1},b_{2},b_{3}\right]$. The syndromes (the new messages) are given by

$$\begin{array}{cc} s_{1} & =W_{1}H^{T}=\left[\begin{array}{cc} a_{1}+a_{2} & a_{1}+a_{3} \end{array}\right] \end{array}$$

(70) $$\begin{array}{cc} \; & ≜\left[\begin{array}{cc} {\bar{a}}_{1} & {\bar{a}}_{2} \end{array}\right], \end{array}$$

$$\begin{array}{cc} s_{2} & =W_{2}H^{T}=\left[\begin{array}{cc} b_{1}+b_{2} & b_{1}+b_{3} \end{array}\right] \end{array}$$

(71) $$\begin{array}{cc} \; & ≜\left[\begin{array}{cc} {\bar{b}}_{1} & {\bar{b}}_{2} \end{array}\right]. \end{array}$$

Assume θ=1. Since $\left\lceil \bar{L}\right\rceil =N^{K-1}$, we can apply a *non-symmetric* PIR scheme [34] to decode $s_{1}$. This scheme is shown in Table 2, and has a download cost of $\bar{D}=3$ bits, which is optimal in this case since it meets the converse bound.

The repetition code used in this example is a *perfect code.* While this makes $\bar{L}$ an integer, and meets the converse bound, perfect codes are scarce. In the next example, we show how the proposed scheme performs with non-perfect codes.

#### 5.1.2. L=5, N=2, K=2, f=1, and r=0

In this example, we have $\bar{L}=\log_{2}\left(1+5\right)=2.58$, and C=2/3. We show that $\bar{D}=\left\lceil \left\lceil \bar{L}\right\rceil /C\right\rceil =5$ bits is achievable. As in the previous example, we start by constructing a [5,2,3] linear block code. Differently though, this is not a repetition code, and is characterized by

(72) $$\begin{array}{c} G=\left[\begin{array}{ccccc} 1 & 0 & 1 & 1 & 1\\ 0 & 1 & 1 & 1 & 0 \end{array}\right],\;H=\left[\begin{array}{ccccc} 1 & 1 & 1 & 0 & 0\\ 1 & 1 & 0 & 1 & 0\\ 1 & 0 & 0 & 0 & 1 \end{array}\right]. \end{array}$$

The syndromes s have length $\left\lceil \bar{L}\right\rceil$. Specifically,

$$\begin{array}{cc} s_{1} & =W_{1}H^{T}=\left[\begin{array}{ccc} a_{1}+a_{2}+a_{3} & a_{1}+a_{2}+a_{4} & a_{1}+a_{5} \end{array}\right] \end{array}$$

(73) $$\begin{array}{cc} \; & ≜\left[\begin{array}{ccc} {\bar{a}}_{1} & {\bar{a}}_{2} & {\bar{a}}_{3} \end{array}\right], \end{array}$$

$$\begin{array}{cc} s_{2} & =W_{2}H^{T}=\left[\begin{array}{ccc} b_{1}+b_{2}+b_{3} & b_{1}+b_{2}+b_{4} & b_{1}+b_{5} \end{array}\right] \end{array}$$

(74) $$\begin{array}{cc} \; & ≜\left[\begin{array}{ccc} {\bar{b}}_{1} & {\bar{b}}_{2} & {\bar{b}}_{3} \end{array}\right]. \end{array}$$

Since $\left\lceil \bar{L}\right\rceil =N^{K-1}+1$, we follow the methodology in [34]; we privately download $N^{K-1}=2$ bits (${\bar{a}}_{1}$ and ${\bar{a}}_{2}$) using the non-symmetric PIR scheme in the previous example, and then privately download the remaining 1 bit (${\bar{a}}_{3}$) using the scheme in [37]. The technique in [37] in this case is such that the user requests random linear combinations of $\left[{\bar{a}}_{3}\;{\bar{b}}_{3}\right]$ from database 1 using a random binary vector *h*, and the same from database 2 yet with $h^{\prime }=h+e_{\theta }$, where $e_{i}$ is the *i*th standard basis vector. The full PIR scheme is shown in Table 3, and it has a download cost of $\bar{D}=5$ bits, which is 1 bit away from the converse bound since the code used is non-perfect.

### 5.2. The General Scheme with Caching

For general *L*, *N*, *K*, and *f*, we construct an $\left[L,L-\left\lceil \bar{L}\right\rceil ,2f+1\right]$ linear block code. From the Gilbert–Varshamov bound [33], we know that such a code exists if

(75) 2L¯≤∑j=02fLj.

In addition, such a code must satisfy the Hamming bound [33]:

(76) ∑j=0fLj≤2L¯.

By the definition of $\bar{L}$ in (9), both (75) and (76) are satisfied, and so the code exists and is able to correct *f* bit flips.

Next, we map each message to its corresponding syndrome of the constructed code, which is of length $L-\left(L-\left\lceil \bar{L}\right\rceil \right)=\left\lceil \bar{L}\right\rceil$. The user then retrieves the syndrome $s_{\theta }$ according to a PIR scheme with *N* databases, *K* messages, and $\left\lceil \bar{L}\right\rceil$ message length. For the case r=0, by ([34], Theorem 1), a download cost of $\left\lceil \left\lceil \bar{L}\right\rceil /C\right\rceil$ is achievable in this case. Finally, correctness is guaranteed since querying for the syndrome $s_{\theta }$ allows the user to decode $W_{\theta }$ as the unique word in the syndrome’s coset with the least Hamming distance from ${\hat{W}}_{\theta }$ [32]. This shows that (22) holds specifically when r=0.

For the case when r≠0, the user will have access to cached linear combinations of $W_{i}$ for all i∈[K]. These cached linear combinations are given by $W_{i}R_{i}$, where $R_{i}$ is a matrix of dimension $\left(L\times \left\lceil \bar{L}\right\rceil \right)$. For the purposes of our cache-aided achievability, we let

(77) $$\begin{array}{c} R_{i}=H^{T},\;\forall i\in \left[K\right], \end{array}$$

where H is the parity check matrix of the code. *This means that during the prefetching phase, bits from our desired syndrome are being cached,* and what is left to download is the remaining $\left\lceil \bar{L}\right\rceil -Lr$ bits.

To this end, we develop some novel schemes for cache-aided PIR with an arbitrary message length that utilize the results from [29]. In particular, for all s∈{1,2,…,K−1}, we define the message length of a cache-aided PIR scheme from [29] with caching ratio $r_{s}$ as

(78) Lr(s)=K−2s−1+∑i=0K−1−sK−1s+i(N−1)iN,

and the normalized download cost of such a scheme as

(79) Dr(s)=∑i=0K−1−sKs+1+i(N−1)iNK−2s−1+∑i=0K−1−sK−1s+i(N−1)iN.

For very low caching ratio *r*, we recall from [29] that the optimal normalized download cost of a cache-aided PIR scheme is

(80) $$\begin{array}{c} D^{*}\left(r\right)=\left(1-r\right)\cdot \sum_{i=0}^{K-1}\frac{1}{N^{i}}-r\cdot \sum_{i=0}^{K-2}\frac{K-1-i}{N^{i}}, \end{array}$$

and that for very high caching ratio *r* (in the context of this work), the optimal normalized download cost of a cache-aided PIR scheme is

(81) $$\begin{array}{c} D^{*}\left(r\right)=\left(1-r\right). \end{array}$$

With these tools in hand, in the remainder of this section, we describe our achievable schemes for very low and very high caching ratios for cache-aided PIR with arbitrary message length, and show that they achieve the download costs in Theorem 2.

### 5.3. Very Low Caching Ratio: Proof of (22)

What follows is a cache-aided achievable scheme for retrieving an arbitrary *L* bits for very low caching ratios ($0<r\leq r_{1}=\frac{1}{1+N+N^{2}+\cdots +N^{K-1}}$). We first use an optimal cache-aided PIR scheme with message size $L_{r}\left(1\right)$. Within the desired *L* bits (including the cached bits), we view each $L_{r}\left(1\right)$ bits as a group, and proceed until the number of desired bits remaining is strictly less than $L_{r}\left(1\right)$. To this end, we have

(82) $$\begin{array}{c} L=G_{0}L_{r}\left(1\right)+L_{0}, \end{array}$$

where $G_{0}=\left\lfloor \frac{L}{L_{r}\left(1\right)}\right\rfloor$ and $0\leq L_{0}\leq L_{r}\left(1\right)-1$. If $L_{0}=0$, then the retrieval is completed. If not, then for the $L_{0}$ bits that remain, we use an optimal asymmetric PIR scheme with message size $N^{K-1}$ (without caching). Within the remaining $L_{0}$ desired bits, we view each $N^{K-1}$ bits as a group, and proceed until the number of desired bits remaining is strictly less than $N^{K-1}$. To this end, we have

(83) $$\begin{array}{c} L_{0}=G_{1}N^{K-1}+L_{1}, \end{array}$$

where $G_{1}=\left\lfloor \frac{L_{0}}{N^{k-1}}\right\rfloor$ and $0\leq L_{1}\leq N^{K-1}-1$. If $L_{1}=0$, then the retrieval is completed. If not, then for the $L_{1}$ bits that remain, we use the scheme in [37] with *N* databases and message size N−1. Within the remaining $L_{1}$ bits, we view each N−1 bits as a group, and proceed until the number of desired bits remaining is strictly less than N−1. To this end, we have

(84) $$\begin{array}{c} L_{1}=G_{2}\left(N-1\right)+L_{2}, \end{array}$$

where $G_{2}=\left\lfloor \frac{L_{1}}{N-1}\right\rfloor$ and $0\leq L_{2}\leq N-2$. If $L_{2}=0$, then the retrieval is completed. If $L_{2}$ bits still remain, we use the scheme in [37] with $L_{2}+1$ databases and message size $L_{2}$. Therefore, the message size and the achievable download cost are

(85) $$\begin{array}{cc} L & =G_{0}L_{r}\left(1\right)+G_{1}N^{K-1}+G_{2}\left(N-1\right)+L_{2}, \end{array}$$

(86) $$\begin{array}{cc} D & =\left\{\begin{array}{cc} G_{0}L_{r}\left(1\right)D^{*}\left(r_{1}\right)+G_{1}\frac{N^{K-1}}{C}+G_{2}N, & \mathrm{if}\;L_{2}=0,\\ G_{0}L_{r}\left(1\right)D^{*}\left(r_{1}\right)+G_{1}\frac{N^{K-1}}{C}+G_{2}N+L_{2}+1, & \mathrm{otherwise}. \end{array}\right. \end{array}$$

We next show that the achievable download cost in (86) satisfies $D\leq \lceil D^{*}\left(r\right)\cdot L\rceil$. To this end, we have the following lemma.

**Lemma 3.**  *For two very low caching ratios $r_{a}$ and $r_{b}$ with $0\leq r_{a}\leq r_{b}\leq r_{1}$, we have*

(87) $$\begin{array}{c} D^{*}\left(r_{a}\right)-D^{*}\left(r_{b}\right)=\left(r_{b}-r_{a}\right)\cdot D_{c}, \end{array}$$

*where $D_{c}=\sum_{i=0}^{K-1}\frac{K-i}{N^{i}}$.*

**Proof.**  We begin from the left-hand side of (87) and use (80) to write

$$\begin{array}{cc} \; & D^{*}\left(r_{a}\right)-D^{*}\left(r_{b}\right) \end{array}$$

(88) $$\begin{array}{cc} \; & =\left(\left(1-r_{a}\right)\cdot \sum_{i=0}^{K-1}\frac{1}{N^{i}}-r_{a}\cdot \sum_{i=0}^{K-2}\frac{K-1-i}{N^{i}}\right)-\left(\left(1-r_{b}\right)\cdot \sum_{i=0}^{K-1}\frac{1}{N^{i}}-r_{b}\cdot \sum_{i=0}^{K-2}\frac{K-1-i}{N^{i}}\right) \end{array}$$

(89) $$\begin{array}{cc} \; & =\left(r_{b}-r_{a}\right)\cdot \sum_{i=0}^{K-1}\frac{1}{N^{i}}+\left(r_{b}-r_{a}\right)\cdot \sum_{i=0}^{K-2}\frac{K-1-i}{N^{i}} \end{array}$$

(90) $$\begin{array}{cc} \; & =\left(r_{b}-r_{a}\right)\cdot \sum_{i=0}^{K-1}\frac{1+(K-1-i)}{N^{i}} \end{array}$$

(91) $$\begin{array}{cc} \; & =\left(r_{b}-r_{a}\right)\cdot \sum_{i=0}^{K-1}\frac{K-i}{N^{i}}. \end{array}$$

Defining $D_{c}=\sum_{i=0}^{K-1}\frac{K-i}{N^{i}}$ concludes the proof. □

Now towards proving $D\leq \lceil D^{*}\left(r\right)\cdot L\rceil$, it suffices to show that $D<D^{*}\left(r\right)\cdot L+1$ for two cases. For the first case, let $L_{2}=0$. We wish to show that

$$\begin{array}{cc} \; & G_{0}L_{r}\left(1\right)D^{*}\left(r_{1}\right)+G_{1}\frac{N^{K-1}}{C}+G_{2}N+L_{2} \end{array}$$

(92) $$\begin{array}{cc} \; & <\;D^{*}\left(r\right)\;\cdot \;\left(G_{0}L_{r}\left(1\right)\;+\;G_{1}N^{K-1}\;+\;G_{2}\left(N\;-\;1\right)\;+\;L_{2}\right)\;+\;1. \end{array}$$

First, we group the terms in (92); we need to show that

$$\begin{array}{cc} \; & -G_{0}L_{r}\left(1\right)\;\cdot \;\left(D^{*}\left(r\right)-D^{*}\left(r_{1}\right)\right)+G_{1}N^{K-1}\;\cdot \;\left(\frac{1}{C}-D^{*}\left(r\right)\right)-\left(G_{2}\left(N-1\right)+L_{2}\right)D^{*}\left(r\right) \end{array}$$

(93) $$\begin{array}{cc} \; & \;<1-G_{2}N-L_{2}. \end{array}$$

Focusing on the left-hand side of (93), we use Lemma 3 to simplify the expression, while noting that $D^{*}\left(0\right)=\frac{1}{C}$, as follows:

$$\begin{array}{cc} \; & -G_{0}L_{r}\left(1\right)\cdot \left(D^{*}\left(r\right)-D^{*}\left(r_{1}\right)\right)+G_{1}N^{K-1}\cdot \left(\frac{1}{C}-D^{*}\left(r\right)\right)-\left(G_{2}\left(N-1\right)+L_{2}\right)D^{*}\left(r\right) \end{array}$$

(94) $$\begin{array}{cc} \; & =-G_{0}L_{r}\left(1\right)D_{c}\left(r_{1}-r\right)+G_{1}N^{K-1}D_{c}r-\left(G_{2}\left(N-1\right)+L_{2}\right)\left(\frac{1}{C}-D_{c}r\right) \end{array}$$

(95) $$\begin{array}{cc} \; & =D_{c}\cdot \left(-G_{0}L_{r}\left(1\right)r_{1}+G_{0}L_{r}\left(1\right)r+G_{1}N^{K-1}r+G_{2}\left(N-1\right)r+L_{2}r\right)-\frac{G_{2}\left(N-1\right)+L_{2}}{C} \end{array}$$

(96) $$\begin{array}{cc} \; & =D_{c}\cdot \left(-G_{0}L_{r}\left(1\right)r_{1}+Lr\right)-\frac{G_{2}\left(N-1\right)+L_{2}}{C} \end{array}$$

(97) $$\begin{array}{cc} \; & =D_{c}\cdot \left(-G_{0}+Lr\right)-\frac{G_{2}\left(N-1\right)+L_{2}}{C}. \end{array}$$

Note that Lr is the number of cached bits, and that $G_{0}$ is the number of times a cache-aided PIR scheme is used. For very low caching ratios, these quantities are equal, and so we have

(98) $$\begin{array}{c} D_{c}\cdot \left(Lr-G_{0}\right)-\frac{G_{2}\left(N-1\right)+L_{2}}{C}=-\frac{G_{2}\left(N-1\right)+L_{2}}{C}. \end{array}$$

Now, substituting (98) back into (93), we now need to show

(99) $$\begin{array}{c} 0<1-G_{2}N-L_{2}+\frac{G_{2}\left(N-1\right)+L_{2}}{C}. \end{array}$$

If N=1, then $G_{2}=0$, and so (99) clearly follows. For the case when N≥2, plugging in $C=\frac{N^{K-1}\left(N-1\right)}{N^{K}-1}$ to the right-hand side of (99) gives

(100) $$\begin{array}{cc} \; & 1-G_{2}N-L_{2}+\frac{G_{2}\left(N-1\right)+L_{2}}{C} \end{array}$$

$$\begin{array}{cc} \; & =1-G_{2}N+G_{2}\frac{N^{K}-1}{N^{K-1}}+L_{2}\left(\frac{N^{K}-1}{N^{K-1}\left(N-1\right)}-1\right) \end{array}$$

(101) $$\begin{array}{cc} \; & =1-G_{2}\frac{1}{N^{K-1}}+L_{2}\left(\frac{N^{K-1}-1}{N^{K-1}\left(N-1\right)}\right). \end{array}$$

We wish to find a lower bound for (101). To this end, we want to maximize $G_{2}$ and minimize $L_{2}$. We know that $L_{2}\geq 1$, but this also means that $G_{2}\left(N-1\right)<L_{1}\leq N^{K-1}-1$ from (84). Plugging these values into (101) gives

$$\begin{array}{cc} \; & 1-G_{2}\frac{1}{N^{K-1}}+L_{2}\left(\frac{N^{K-1}-1}{N^{K-1}\left(N-1\right)}\right) \end{array}$$

(102) $$\begin{array}{cc} \; & \geq 1-\frac{G_{2}\left(N-1\right)}{N^{K-1}\left(N-1\right)}+\frac{N^{K-1}-1}{N^{K-1}\left(N-1\right)} \end{array}$$

(103) $$\begin{array}{cc} \; & >1-\frac{N^{K-1}-1}{N^{K-1}\left(N-1\right)}+\frac{N^{K-1}-1}{N^{K-1}\left(N-1\right)}=1. \end{array}$$

and so (99) holds for N≥2.

For the second case, let $L_{2}\geq 1$. We wish to show that

$$\begin{array}{cc} \; & G_{0}L_{r}\left(1\right)D^{*}\left(r_{1}\right)+G_{1}\frac{N^{K-1}}{C}+G_{2}N+L_{2}+1 \end{array}$$

(104) $$\begin{array}{cc} \; & <D^{*}\left(r\right)\cdot \left(G_{0}L_{r}\left(1\right)+G_{1}N^{K-1}+G_{2}\left(N-1\right)+L_{2}\right)+1. \end{array}$$

First, we group the terms in (104); we need to show that

$$\begin{array}{cc} \; & G_{1}N^{K-1}\cdot \left(\frac{1}{C}-D^{*}\left(r\right)\right)-G_{0}L_{r}\left(1\right)\cdot \left(D^{*}\left(r\right)-D^{*}\left(r_{1}\right)\right)-\left(G_{2}\left(N-1\right)+L_{2}\right)D^{*}\left(r\right) \end{array}$$

(105) $$\begin{array}{cc} \; & <1-G_{2}N-L_{2}-1. \end{array}$$

By (98), we substitute the left-hand side of (105) so that we have

(106) $$\begin{array}{c} 0<1-G_{2}N-L_{2}+\frac{G_{2}\left(N-1\right)+L_{2}}{C}-1. \end{array}$$

Since $L_{2}\geq 1$, we have N≥2, and so (106) holds by (103). This completes the proof that $D\leq \lceil D^{*}\left(r\right)\cdot L\rceil$ for very low caching ratios.

Since the above PIR scheme is constructed as a concatenation of several PIR schemes that are both correct and private, by ([34], Theorem 4), the above scheme is both correct and private. To conclude our proof, we define a normalized version of *r*:

(107) $$\begin{array}{c} \tilde{r}=\frac{Lr}{\left\lceil \bar{L}\right\rceil }, \end{array}$$

as the *effective* caching ratio. Clearly, by (13), $0\leq \tilde{r}\leq 1$. Now, since the above PIR scheme retrieves *L* bits (including cached bits) at a download cost of $D\leq \lceil D^{*}\left(r\right)\cdot L\rceil$, this scheme can be used to retrieve $\left\lceil \bar{L}\right\rceil$ bits (including some Lr cached bits) at a download cost of $\bar{D}\leq \left\lceil D^{*}\left(\tilde{r}\right)\cdot \left\lceil \bar{L}\right\rceil \right\rceil$. Expanding this statement gives

(108) $$\begin{array}{cc} \; & \bar{D}\leq \left\lceil D^{*}\left(\tilde{r}\right)\cdot \left\lceil \bar{L}\right\rceil \right\rceil \end{array}$$

(109) $$\begin{array}{cc} \; & =\left\lceil \left\lceil \bar{L}\right\rceil \left(1-\tilde{r}\right)\cdot \;\sum_{i=0}^{K-1}\frac{1}{N^{i}}-\left\lceil \bar{L}\right\rceil \tilde{r}\cdot \;\sum_{i=0}^{K-2}\frac{K-1-i}{N^{i}}\right\rceil \end{array}$$

(110) $$\begin{array}{cc} \; & =\left\lceil \left(\left\lceil \bar{L}\right\rceil -Lr\right)\cdot \sum_{i=0}^{K-1}\frac{1}{N^{i}}-Lr\cdot \sum_{i=0}^{K-2}\frac{K-1-i}{N^{i}}\right\rceil , \end{array}$$

which is precisely (22).

### 5.4. Very High Caching Ratio: Proof of (23)

What follows is a cache-aided achievable scheme for retrieving an arbitrary *L* bits, for very high caching ratios ($r_{K-1}=\frac{1}{1+N}\leq r\leq 1$). In this scheme, we only use an optimal cache-aided PIR scheme with message size $L_{r}\left(K-1\right)=1+N$. We note that in this scheme, for each bit we have cached, we can download 1 bit from each of the *N* databases to get a total of *N* unknown bits at a download cost of *N* bits.

Within the desired *L* bits (including cached bits), we view each $L_{r}\left(K-1\right)$ bits as a group, and proceed until the number of desired and *unknown* L−Lr bits remaining is strictly less than *N*. To this end, we have

(111) $$\begin{array}{c} L=G_{0}L_{r}\left(K-1\right)+L_{0}, \end{array}$$

where $G_{0}=\left\lfloor \frac{L-Lr}{N}\right\rfloor$, and $L_{0}=L-G_{0}L_{r}\left(K-1\right)$. We define $C_{0}=Lr-G_{0}$ as the number of *unused* cached bits thus far in our scheme. If we have $L_{0}=C_{0}$, then we have all of our desired information, and we are done. Otherwise, we still have $L_{0}-C_{0}<N$ bits left to download. Since the caching ratio *r* is very high, we have $C_{0}\geq 1$, and so we can use this bit, as noted above, to download 1 bit from $L_{0}-C_{0}<N$ databases each to obtain the remaining $L_{0}-C_{0}$ unknown bits at a download cost of $L_{0}-C_{0}$ bits. Therefore, the message size and the achievable download cost are

(112) $$\begin{array}{cc} L & =G_{0}L_{r}\left(K-1\right)+L_{0}, \end{array}$$

(113) $$\begin{array}{cc} D & =G_{0}L_{r}\left(K-1\right)D^{*}\left(r_{K-1}\right)+L_{0}-C_{0}. \end{array}$$

We next show that the achievable download cost in (113) satisfies $D\leq \lceil D^{*}\left(r\right)\cdot L\rceil$. To this end, it it suffices to show that $D<D^{*}\left(r\right)\cdot L+1$, or more specifically, that

(114) $$\begin{array}{cc} \; & G_{0}L_{r}\left(K\;-\;1\right)D^{*}\left(r_{K-1}\right)\;+\;L_{0}\;-\;C_{0}<D^{*}\left(r\right)\cdot L\;+\;1. \end{array}$$

First, we rearrange the terms in (114) as

(115) $$\begin{array}{cc} \; & G_{0}L_{r}\left(K\;-\;1\right)D^{*}\left(r_{K-1}\right)\;+\;L_{0}\;-\;C_{0}\;-\;D^{*}\left(r\right)\cdot L<1, \end{array}$$

and then we reduce the left-hand side of (115) as follows

$$\begin{array}{cc} \; & G_{0}L_{r}\left(K-1\right)D^{*}\left(r_{K\;-\;1}\right)\;+\;L_{0}\;-\;C_{0}\;-\;D^{*}\left(r\right)\cdot L \end{array}$$

(116) $$\begin{array}{cc} \; & =G_{0}\left(1+N\right)\left(1-\frac{1}{1+N}\right)+L_{0}-C_{0}-\left(1-r\right)\cdot L \end{array}$$

(117) $$\begin{array}{cc} \; & =G_{0}N+L_{0}-C_{0}-L+Lr \end{array}$$

(118) $$\begin{array}{cc} \; & =-C_{0}-G_{0}+Lr=0. \end{array}$$

Thus, (114) holds, and so this completes the proof that $D\leq \lceil D^{*}\left(r\right)\cdot L\rceil$ for very high caching ratios.

Again, since the above PIR scheme is constructed as a concatenation of several PIR schemes that are both correct and private, by ([34], Theorem 4), the above scheme is both correct and private. Furthermore, since the above PIR scheme retrieves *L* bits (including cached bits) at a download cost of $D\leq \lceil D^{*}\left(r\right)\cdot L\rceil$, this scheme can be used to retrieve $\left\lceil \bar{L}\right\rceil$ bits (including some Lr cached bits) at a download cost of $\bar{D}\leq \left\lceil D^{*}\left(\tilde{r}\right)\cdot \left\lceil \bar{L}\right\rceil \right\rceil$. Expanding this statement gives

(119) $$\begin{array}{cc} \bar{D} & \leq \left\lceil D^{*}\left(\tilde{r}\right)\cdot \left\lceil \bar{L}\right\rceil \right\rceil \end{array}$$

(120) $$\begin{array}{cc} \; & =\left\lceil \left(1-\tilde{r}\right)\cdot \left\lceil \bar{L}\right\rceil \right\rceil \end{array}$$

(121) $$\begin{array}{cc} \; & =\left\lceil \left\lceil \bar{L}\right\rceil -Lr\right\rceil =\left\lceil \bar{L}\right\rceil -Lr, \end{array}$$

which is precisely (23).

## 6. Proof of Theorem 3: Achievability for K=3 with Mid-Range Caching Ratios

What follows is a cache-aided achievable scheme for retrieving an arbitrary *L* bits, for mid-range caching ratios given fixed K=3 setting $\left(\frac{1}{1+N+N^{2}}=r_{1}<r<r_{2}=\frac{1}{1+N}\right)$. This scheme leverages cache-aided PIR schemes for very high and very low caching ratios but within an asymmetric PIR setting instead.

First, consider the asymmetric cache-aided PIR scheme with N=3 and L=3 in Table 4. This scheme does not utilize all of the databases, nor does it utilize the cache in full. This scheme downloads one useful bit privately at a cost of 1 bit, and it is an asymmetric version of the cache-aided PIR scheme for very high caching ratios. This scheme can be repeated up to five more times to get up to five more useful bits, and each additional bit is obtained privately.

Next, consider the asymmetric cache-aided PIR scheme with N=3 and L=6 in Table 5. While this scheme does utilize all of the databases, it has asymmetric traffic between the databases, and it also does not utilize the cache in full. This scheme downloads 1+N useful bits at a cost of 2+N, and it is an asymmetric version of the cache-aided PIR scheme for very low caching ratios. Once again, this scheme can be repeated up to five more times to get up to 5·(1+N) more useful bits, and each additional set of 1+N bits is obtained privately.

In these examples, we see that each scheme can be used a total of N·Lr=6 times. Now, note that these two schemes can be used *in conjunction* with one another, and that rather than repeating the same scheme over and over again, we can just use them interchangeably to suit our needs.

Consider a cache-aided PIR example where N=3, L=14, and $r=\frac{2}{14}$. Note that *r* is now mid-range. We can use a combination of the asymmetric very high caching ratio scheme and very low caching ratio scheme to download the remaining 12 useful bits as shown in Table 6. First, we use the asymmetric very high caching ratio scheme four times to obtain four useful bits at a cost of 4 bits total. Then, we use the the asymmetric very low caching ratio scheme two times to download the remaining 2·(1+N)=8 useful bits at a cost of 2·(2+N)=10, and so the total download cost is 14.

It is also worth noting that in the same scenario, but with L=13 and $r=\frac{2}{13}$, we can use almost the almost the same query structure as in Table 6. The only difference is that we *truncate* the given scheme by not making the query for $a_{14}$. In this particular case, this truncation strategy can be performed again to obtain an L=12, $r=\frac{2}{12}$ query structure.

In general, one can use a combination of N·Lr−1 very high and very low caching ratio schemes, and then if the remaining number of useful bits left to download is some *ℓ* with 1<l<N+1, use a truncated very low caching ratio scheme. Otherwise, just a normal very high or very low caching ratio scheme can be used.

In order to determine the number of times these very high and very low schemes are used, along with the number of bits that are downloaded via the truncation strategy, we define three terms as follows:

(122) $$\begin{array}{cc} G_{1} & =\left\lfloor \frac{L_{r}\left(1\right)\cdot Lr-L}{N}\right\rfloor , \end{array}$$

(123) $$\begin{array}{cc} G_{2} & =\left\lfloor \frac{L-L_{r}\left(2\right)\cdot Lr}{N}\right\rfloor , \end{array}$$

(124) $$\begin{array}{cc} L_{3} & =L-(G_{1}+G_{2}\left(1+N\right))-Lr. \end{array}$$

The $G_{1}$ term is the number of times a very high caching ratio scheme is used, while $G_{2}$ is the number of times a very low caching ratio scheme is used. The $L_{3}$ term is the number of bits obtained from the truncation strategy when it is used. According to these terms, it follows that the message size and the achievable download cost are

(125) $$\begin{array}{cc} L & =G_{1}+G_{2}\left(1+N\right)+L_{3}+Lr, \end{array}$$

(126) $$\begin{array}{cc} D & =\left\{\begin{array}{cc} G_{1}+G_{2}\left(2+N\right), & \mathrm{if}\;L_{3}=0,\\ G_{1}+G_{2}\left(2+N\right)+L_{3}+1, & \mathrm{otherwise}. \end{array}\right. \end{array}$$

Lastly, for mid-range caching ratios with K=3, we recall from [29] that the optimal normalized download cost of a cache-aided PIR scheme is

(127) $$\begin{array}{c} D^{*}\left(r\right)=\left(1-r\right)\left(1+\frac{1}{N}\right)-r. \end{array}$$

We next show that the achievable download cost in (126) satisfies $D\leq \left\lceil D^{*}\left(r\right)\cdot L\right\rceil$. To this end, it suffices to show that $D<D^{*}\left(r\right)\cdot L+1$ for two cases. For the first case, let $L_{3}=0$. We wish to show that

(128) $$\begin{array}{cc} \; & G_{1}+G_{2}\left(2+N\right)+L_{3}-D^{*}\left(r\right)\cdot L<1. \end{array}$$

Reducing the left-hand side of (128), we have

$$\begin{array}{cc} \; & G_{1}+G_{2}\left(2+N\right)+L_{3}-D^{*}\left(r\right)\cdot L \end{array}$$

(129) $$\begin{array}{cc} \; & =G_{1}\;+\;G_{2}\left(2\;+\;N\right)\;+\;L_{3}\;-\;\left(\left(1\;-\;r\right)\;\cdot \;\left(1\;+\;\frac{1}{N}\right)\;-\;r\right)\cdot L \end{array}$$

(130) $$\begin{array}{cc} \; & =G_{1}+G_{2}\left(2+N\right)+L_{3}-\;\left(1\;-\;2r\;+\;\frac{1\;-\;r}{N}\right)\;\cdot \;\left(G_{1}\;+\;G_{2}\left(1\;+\;N\right)\;+\;L_{3}\;+\;Lr\right) \end{array}$$

(131) $$\begin{array}{cc} \; & =G_{2}-Lr+\left(2r-\frac{1-r}{N}\right)\cdot L \end{array}$$

(132) $$\begin{array}{cc} \; & =G_{2}+Lr-\frac{L-Lr}{N} \end{array}$$

(133) $$\begin{array}{cc} \; & =G_{2}-\frac{L-(1+N)\cdot Lr}{N} \end{array}$$

(134) $$\begin{array}{cc} \; & =G_{2}-\frac{L-L_{r}\left(2\right)\cdot Lr}{N}. \end{array}$$

Substituting (134) into (128), we need to show that

(135) $$\begin{array}{c} G_{2}-\frac{L-L_{r}\left(2\right)\cdot Lr}{N}<1, \end{array}$$

which clearly holds by (123). It follows that (128) holds when $L_{3}=0$. To show that this is also the case when $L_{3}\geq 1$, we use a lemma.

**Lemma 4.**  *In the K=3 setting, for any caching ratio r with $\frac{1}{1+N+N^{2}}=r_{1}<r<r_{2}=\frac{1}{1+N}$, we have*

(136) $$\begin{array}{c} L_{3}=0⟺\frac{L-L_{r}\left(2\right)\cdot Lr}{N}\in Z \end{array}$$

The proof of Lemma 4 can be found in Appendix B.

Now, for the second case, let $L_{3}\geq 1$. We wish to show that

(137) $$\begin{array}{cc} \; & G_{1}+G_{2}\left(2+N\right)+L_{3}-D^{*}\left(r\right)\cdot L<0. \end{array}$$

By (134), we substitute the left-hand side of (137) so that we have

(138) $$\begin{array}{c} G_{2}-\frac{L-L_{r}\left(2\right)\cdot Lr}{N}<0, \end{array}$$

which holds by Lemma 4. Thus, this completes the proof that $D\leq \left\lceil D^{*}\left(r\right)\cdot L\right\rceil$ for mid-range caching ratios in the K=3 setting.

Since the above PIR scheme is constructed as a concatenation of several PIR schemes that are both correct and private (by [34], Theorem 4), the above scheme is both correct and private. Furthermore, since the above PIR scheme retrieves *L* bits (including cached bits) at a download cost of $D\leq \lceil D^{*}\left(r\right)\cdot L\rceil$, this scheme can be used to retrieve $\left\lceil \bar{L}\right\rceil$ bits (including some Lr cached bits) at a download cost of $\bar{D}\leq \left\lceil D^{*}\left(\tilde{r}\right)\cdot \left\lceil \bar{L}\right\rceil \right\rceil$. Expanding this statement gives

(139) $$\begin{array}{cc} \bar{D} & \leq \left\lceil D^{*}\left(\tilde{r}\right)\cdot \left\lceil \bar{L}\right\rceil \right\rceil \end{array}$$

(140) $$\begin{array}{cc} \; & =\left\lceil \left\lceil \bar{L}\right\rceil \left(1-\tilde{r}\right)\left(1+\frac{1}{N}\right)-\left\lceil \bar{L}\right\rceil \tilde{r}\right\rceil \end{array}$$

(141) $$\begin{array}{cc} \; & =\left\lceil \left(\left\lceil \bar{L}\right\rceil -Lr\right)\left(1+\frac{1}{N}\right)-Lr\right\rceil \end{array}$$

which is precisely (26).

## 7. Discussion

As seen in Corollary 1, for very low and very high effective caching ratios, we obtain full characterizations of the optimal download cost ${\bar{D}}_{L}$ for fixed L,N,K, and *f*. What remains is to perform the same for an effective caching ratio $\tilde{r}$, defined in (107), with $\frac{1}{1+N+N^{2}+\cdots +N^{K-1}}=r_{1}\leq \tilde{r}\leq r_{K-1}=\frac{1}{1+N}$, i.e., such caching ratios that are *mid-range*. With Theorem 3 and Corollary 2, this has been performed for the K=3 case. However, this is still an open question for when *K* is arbitrary.

Our approach for our achievability when $\tilde{r}\neq 0$ has been to describe an arbitrary message length PIR scheme for a setting with unknown prefetching, and then show that the download cost *D* of such a scheme satisfies $D\leq \left\lceil D^{*}\left(\tilde{r}\right)\cdot \left\lceil \bar{L}\right\rceil \right\rceil$. This approach mirrors what was performed in [34] for the classical PIR setting.

From [29], for $r_{s}<r<r_{s+1}$ and α∈[0,1] with $r=\alpha r_{s}+\left(1-\alpha \right)r_{s+1}$, we define

(142) $$\begin{array}{c} \bar{D}\left(r\right)=\alpha D_{r}\left(s\right)+\left(1-\alpha \right)D_{r}\left(s+1\right). \end{array}$$

We know that $\bar{D}\left(r\right)=D^{*}\left(r\right)$ for very low and very high caching ratio *r*, and this is used in our approach for Theorem 2. This is likewise the case for mid-range caching ratios *r* when K=3 in Theorem 3. For when $\bar{D}\left(r\right)\neq D^{*}\left(r\right)$, as is the case for most mid-range caching ratios, we can still attempt to describe a scheme, and show that the download cost $D\leq \left\lceil \bar{D}\left(\tilde{r}\right)\cdot \left\lceil \bar{L}\right\rceil \right\rceil$ to obtain some useful result.

Our goal in this section is to present a motivating example that shows what these results may look like. Consider the following example setting: N=3, K=4, and $r_{K-2}\leq r\leq r_{K-1}$. We have $r_{1}=\frac{1}{40}$ and $r_{K-1}=\frac{1}{4}$, and so a caching ratio is mid-range in this setting if $\frac{1}{40}\leq r\leq \frac{1}{4}$. However, for our purposes, we will focus on the subset of mid-range caching ratios *r* satisfying $r_{K-2}=\frac{2}{17}\leq r\leq \frac{1}{4}$. With this in mind, let us consider some scenarios with a caching ratio $r=\frac{1}{7}$ starting with the case when the number of cached bits is 3, and so the total message length is 21. Using the methods found in this work, we have a scheme satisfying $D\leq \left\lceil \bar{D}\left(\tilde{r}\right)\cdot \left\lceil \bar{L}\right\rceil \right\rceil$ given in Table 7.

Using these same methods, if there are two cached bits with a total message length of 14, then we also have a scheme satisfying $D\leq \left\lceil \bar{D}\left(\tilde{r}\right)\cdot \left\lceil \bar{L}\right\rceil \right\rceil$ using a subset of the queries in Table 7. However, for the case when there is only one cached bit with a total message length of seven, we have no scheme satisfying $D\leq \left\lceil \bar{D}\left(\tilde{r}\right)\cdot \left\lceil \bar{L}\right\rceil \right\rceil$, not with using the methods in this work at least. It is worth noting that for some other mid-range caching ratios with this setting, the scheme from [37] can be used to produce some satisfactory results ($r=\frac{1}{6}$ for example) but not for the case when $r=\frac{1}{7}$ in this setting. This is discussed in more detail in [38].

The question remains: *why does this pattern break, and why it is difficult to find an alternative query structure?* The answer we have come to is that it has not to do with with the value of the *r*, but with *the number number of cached bits Lr.* More specifically, there may be some additional limitation on how low of a download cost can be achieved with a cache-aided arbitrary message length PIR scheme when Lr is relatively low (or in this case, when Lr=1). Investigating such limitations is left to future works.

## 8. Conclusions

In this work, we introduce the cache-aided private updating problem with unknown prefetching, in which a user’s outdated message is to be privately updated by utilizing a private cache and querying a set of replicated and non-colluding databases that have the up-to-date version. To solve this problem, we develop novel *arbitrary message length cache-aided* PIR schemes for different caching ratios. These schemes are then combined with syndrome decoding techniques to guarantee privacy and efficiency. Such schemes are optimal when the system parameters enable the construction of a perfect code according to which the syndrome decoding technique is worked out. In other cases, the achievable download cost has been shown to be within at most 2 bits from a derived converse bound.

Outside of the issues discussed in Section 7, another item that could be resolved in this problem is the inflexible nature of the cache in our achievability. Specifically, the fact that for each i∈[K], we fix $R_{i}=H^{T}$ during the prefetching phase. Imposing less control over the prefetching phase is one direction to be pursued in the research line of cache-aided private updating.

## Figures and Tables

**Table 1:** Key notations and system parameters.

Symbol	Definition
*K*	number of messages
*N*	number of databases
*L*	message length
θ	index of the required message
${\hat{W}}_{\theta }$	outdated message
$W_{\theta }$	current message
*f*	upper bound on differences between outdated and current messages
*Z*	cache content
*ℓ*	number of linearly-combined bits cached from each message
*r*	caching ratio: l/L
$\bar{L}$	number of bits sufficient to update the message: log2(∑i=0fLi)

**Table 2:** Query table for N=K=2, L=3, f=1, and r=0.

Database 1	Database 2
${\bar{a}}_{1}$, ${\bar{b}}_{1}$	${\bar{a}}_{2}+{\bar{b}}_{1}$

**Table 3:** Query table for N=K=2, L=5, f=1, and r=0.

Database 1	Database 2
${\bar{a}}_{1}$, ${\bar{b}}_{1}$	${\bar{a}}_{2}+{\bar{b}}_{1}$
$h_{1}{\bar{a}}_{3}+h_{2}{\bar{b}}_{3}$	$\left(h_{1}+1\right){\bar{a}}_{3}+h_{2}{\bar{b}}_{3}$

**Table 4:** Asymmetric query table with N=K=L=3 and very high *r*. Here, we have $Z=\{a_{1},a_{2},b_{1},b_{2},c_{1},c_{2}\}$.

Database 1	Database 2	Database 3
$a_{3}+b_{1}+c_{1}$		

**Table 5:** Asymmetric query table with N=K=3, L=6 and very low *r*. Here, we have $Z=\{a_{1},a_{2},b_{1},b_{2},c_{1},c_{2}\}$.

Database 1	Database 2	Database 3
$a_{3}+b_{1}$		
$a_{4}+c_{1}$		
$b_{3}+c_{3}$		
	$a_{5}+b_{3}+c_{3}$	$a_{6}+b_{3}+c_{3}$

**Table 6:** Query table for N=K=3, L=14, and mid-range caching ratio $r=\frac{2}{14}$. Here, we have $Z=\{a_{1},a_{2},b_{1},b_{2},c_{1},c_{2}\}$.

Database 1	Database 2	Database 3
$a_{3}+b_{1}+c_{1}$	$a_{4}+b_{1}+c_{1}$	$a_{5}+b_{1}+c_{1}$
		$a_{6}+b_{2}+c_{2}$
$a_{7}+b_{2}$	$a_{11}+b_{2}$	
$a_{8}+c_{2}$	$a_{12}+c_{2}$	
$b_{3}+c_{3}$	$b_{4}+c_{4}$	
	$a_{9}+b_{3}+c_{3}$	$a_{10}+b_{3}+c_{3}$
$a_{13}+b_{4}+c_{4}$		$a_{14}+b_{4}+c_{4}$

**Table 7:** Query table for N=3, K=4, L=21, and $r=\frac{3}{21}$. Here, we have $Z=\{a_{1},a_{2},a_{3},b_{1},b_{2},b_{3},c_{1},c_{2},c_{3},d_{1},d_{2},d_{3}\}$.

Database 1	Database 2	Database 3
$a_{4}+b_{1}+c_{1}$	$a_{7}+b_{1}+c_{1}$	$a_{10}+b_{1}+c_{1}$
$a_{5}+b_{2}+d_{1}$	$a_{8}+b_{2}+d_{1}$	$a_{11}+b_{2}+d_{1}$
$a_{6}+c_{2}+d_{2}$	$a_{9}+c_{2}+d_{2}$	$a_{12}+b_{2}+d_{2}$
$b_{4}+c_{4}+d_{4}$	$b_{5}+c_{5}+d_{5}$	$b_{6}+c_{6}+d_{6}$
$a_{13}+b_{5}+c_{5}+d_{5}$	$a_{15}+b_{4}+c_{4}+d_{4}$	$a_{17}+b_{4}+c_{4}+d_{4}$
$a_{14}+b_{6}+c_{6}+d_{6}$	$a_{16}+b_{6}+c_{6}+d_{6}$	$a_{18}+b_{5}+c_{5}+d_{5}$
$a_{19}+b_{3}+c_{3}+d_{3}$	$a_{20}+b_{3}+c_{3}+d_{3}$	$a_{21}+b_{3}+c_{3}+d_{3}$

## Data Availability

The original contributions presented in this study are included in the article. Further inquiries can be directed to the corresponding author.

## Appendix A. Bound on Effective Value of *f*

For completeness, we show that

(A1) $$\begin{array}{cc} f & <\frac{L}{2}\Leftrightarrow \left\lceil \bar{L}\right\rceil <L, \end{array}$$

and hence if the maximum number of bit flips is more than half the message length, it is optimal to ignore the outdated message (as per Corollary 1’s result).

First, suppose that $f=\left\lfloor \frac{L-1}{2}\right\rfloor <\frac{L}{2}$. If *L* is odd, then $f=\frac{L-1}{2}$ and it follows that

(A2) ∑i=0LLi=2·∑i=0L−12Li=2L⇔∑i=0fLi=2L−1.

So for odd *L*, we have L¯=log2∑i=0fLi=L−1, and so $\frac{L-1}{2}$ is the maximum value of *f* satisfying $\left\lceil \bar{L}\right\rceil <L$ when *L* is odd.

Next, suppose that *L* is even. It follows that

(A3) ∑i=0LLi=2·∑i=0L−12Li+LL2=2L⇔∑i=0fLi<2L−1.

So for even *L*, we have L¯=log2∑i=0fLi<L−1. Also, note that for even *L*,

(A4) ∑i=0L2Li=∑i=0L−12Li+LL2>2L−1.

This means that $\left\lfloor \frac{L-1}{2}\right\rfloor$ is the maximum value of *f* satisfying $\left\lceil \bar{L}\right\rceil <L$ when *L* is even.

Therefore, for any message length *L*, we have the result in Remark 2. This completes the proof.

## Appendix B. Proof of Lemma 4

First, we note that

(A5) $$\begin{array}{cc} \; & \frac{L_{r}\left(1\right)\cdot Lr-L}{N}+\frac{L-L_{r}\left(2\right)\cdot Lr}{N}=N\cdot Lr, \end{array}$$

and so it follows that $G_{1}+G_{2}\in \left\{N\cdot Lr,\;N\cdot Lr-1\right\}$.

Consider the case when $G_{1}+G_{2}=N\cdot Lr$. Plugging this into (124), it can be shown that

(A6) $$\begin{array}{c} G_{2}=\frac{L-L_{r}\left(2\right)\cdot Lr}{N}-\frac{L_{3}}{N}. \end{array}$$

Substituting (A6) back into (124), it can be shown that

(A7) $$\begin{array}{c} G_{1}=\frac{L_{r}\left(1\right)\cdot Lr-L}{N}+\frac{L_{3}}{N}. \end{array}$$

If $L_{3}>0$, then substituting such a value into (A7) would contradict (122). Likewise, if $L_{3}<0$, then substituting such a value into (A6) would contradict (123). Therefore,

(A8) $$\begin{array}{c} G_{1}\;+\;G_{2}=N\;\cdot \;Lr\Rightarrow L_{3}=0\Rightarrow \frac{L-L_{r}\left(2\right)\cdot Lr}{N}\in Z. \end{array}$$

Now consider the case when $G_{1}+G_{2}=N\cdot Lr-1$. Plugging this into (124), it can be shown that

(A9) $$\begin{array}{c} G_{2}=\frac{L-L_{r}\left(2\right)\cdot Lr}{N}-\frac{L_{3}-1}{N}. \end{array}$$

If $L_{3}\leq 0$, then substituting such a value into (A9) would contradict (123). Likewise, if $L_{3}\geq N+1$, then substituting such a value into (A9) would also contradict (123). Therefore, we have

(A10) $$\begin{array}{c} G_{1}\;+\;G_{2}=N\;\cdot \;Lr\;-\;1\Rightarrow \;L_{3}\neq 0\Rightarrow \;\frac{L\;-\;L_{r}\left(2\right)\;\cdot \;Lr}{N}\;\notin \;Z. \end{array}$$

Finally, by combining (A8) and (A10), we can obtain the result in the lemma. This completes the proof.
